# Impact of Stenting with Angioplasty and MTICI 2c-3 Recanalization On Outcome in Acute MCA Occlusion with Underlying Stenosis

**DOI:** 10.1007/s00062-025-01577-6

**Published:** 2025-10-13

**Authors:** Andrea Maria Alexandre, Arturo Consoli, Luca Scarcia, Enrico Di Stasio, Valerio Brunetti, Wen Sun, Yingjie Xu, Xianjun Huang, Charlotte Chung, Alessandro Sgreccia, Mohamad Abdalkader, Nicola Limbucci, Alessandro Pedicelli, Francesco Capasso, Francesco Arba, Ludovica Migliaccio, Mariangela Piano, Maria Porzia Ganimede, Emilio Lozupone, Chiara Gaudino, Francesca Ricchetti, Riccardo Russo, Julien Burel, Francesco D’Argento, Serena Abruzzese, Julien Allard, Nicolas Chausson, Roberta Partesano, Nicola Cavasin, Nicolò Mandruzzato, Joseph Domenico Gabrieli, Pietro Trombatore, Antonio Armando Caragliano, Federico Mazzacane, Giancarlo Salsano, Antioco Sanna, Pietro Panni, Andrea Zini, Frédéric Clarençon, Eytan Raz, Thanh Nguyen, Aldobrando Broccolini

**Affiliations:** 1 Interventional Neuroradiology Unit, Fondazione Policlinico Universitario A. Gemelli IRCCS, Rome, Italy; 2 Department of Diagnostic and Therapeutic Neuroradiology, Hôpital Foch, Suresnes, France; 3 Department of Neuroradiology, Hôpitaux Universitaires Henri-Mondor, Créteil, France; 4 Department of Basic Biotechnological Sciences, Intensive Care and Perioperative Clinics, Fondazione Policlinico Universitario A. Gemelli IRCCS, Rome, Italy; 5 Neurology Unit, Fondazione Policlinico Universitario A. Gemelli IRCCS, Rome, Italy; 6 Department of Neurology, Centre for Leading Medicine and Advanced Technologies of IHM, The First Affiliated Hospital of USTC, Hefei, China; 7 Department of Neurology, First Affiliated Hospital of Wannan Medical College, Wuhu, China; 8 Department of Neurosurgery and Department of Radiology, NYU Langone Health, New York, United States; 9 Department of Radiology and Neurology, Boston Medical Center, Boston, United States; 10 Interventional Neurovascular Unit, Azienda Ospedaliero-Universitaria Careggi, Florence, Italy; 11 Stroke Unit, Azienda Ospedaliero-Universitaria Careggi, Florence, Italy; 12 Department of Neurology and Stroke Center, IRCCS Istituto delle Scienze Neurologiche di Bologna, Bologna, Italy; 13 Neuroradiology Unit, ASST Grande Ospedale Metropolitano Niguarda, Milan, Italy; 14 Neuroradiology Unit, Presidio Ospedaliero Centrale SS Annunziata, Taranto, Italy; 15 Neuroradiology Unit, Ospedale Vito Fazzi, Lecce, Italy; 16 Department of Neuroradiology, Azienda Ospedaliero-Universitaria Policlinico Umberto I, Rome, Italy; 17 Neuroradiology Unit, Azienda Ospedaliera Città della Salute e della Scienza di Torino, Turin, Italy; 18 Department of Radiology, Centre Hospitalier Universitaire de Rouen, Rouen, France; 19 Department of Neuroscience, Catholic University of the Sacred Heart, Rome, Italy; 20 Department of Interventional Neuroradiology, Pitié-Salpêtrière Hospital, Paris, France; 21 Department of Neurology, Centre Hospitalier Sud Francilien, Corbeil-Essonnes, France; 22 Neuroradiology Unit, Azienda Ospedaliero-Universitaria di Parma, Parma, Italy; 23 Neuroradiology Unit, Ospedale dell’Angelo, Mestre, Italy; 24 Interventional Neuroradiology, Azienda Ospedaliera Universitaria Integrata di Verona, Verona, Italy; 25 Neuroradiology Unit, Azienda Ospedaliera di Padova, Padova, Italy; 26 Department of Diagnostic Imaging and of Interventional Radiology and Neuroradiology, Ospedale Garibaldi, Catania, Italy; 27 Neuroradiology Unit, Azienda Ospedaliera Universitaria Policlinico “G. Martino”, Messina, Italy; 28 Department of Stroke Unit and Emergency Neurology, Fondazione Istituto Neurologico Nazionale Casimiro Mondino, Pavia, Italy; 29 Neuroradiology Unit, IRCCS Ospedale Policlinico San Martino, Genova, Italy; 30 Neuroradiology Unit, Ospedale Santa Corona, Pietra Ligure, Italy; 31 Interventional Neuroradiology Unit, IRCCS Ospedale San Raffaele, Milan, Italy

**Keywords:** Ischemic stroke, Large vessel occlusion, Intracranial artery stenosis, Intracranial stenting, Clinical outcome

## Abstract

**Purpose:**

Mechanical thrombectomy (MT) is standard care for acute large vessel occlusion (LVO), but it fails in 10–20% of cases, often due to underlying intracranial artery stenosis (ICAS). In such cases, rescue stenting (RS), with or without angioplasty, may improve recanalization, but its clinical benefit remains debated. The purpose of this study was to define predictors of clinical outcome in this patient population.

**Methods:**

We conducted a retrospective multicenter study including 115 patients with ICAS-related occlusion of the middle cerebral artery (MCA) treated with MT and RS across 27 international stroke centers. Baseline, procedural, and post-procedural variables were analyzed. The outcome measure was the ordinal shift of the 90-day modified Rankin Scale (mRS) score. Stepwise multivariate regression and structural equation modeling (SEM) were used to identify outcome predictors and explore mediation pathways.

**Results:**

Successful recanalization (modified Treatment in Cerebral Infarction (mTICI) score ≥ 2b) was achieved in 94.8% of patients, with 73.0% reaching mTICI 2c‑3. SEM showed that baseline Alberta Stroke Program Early CT Score (ASPECTS), stenting with angioplasty and achieving mTICI 2c‑3 were associated with improved functional outcome, mediated by higher post-procedural ASPECTS. Post-procedural ASPECTS influenced functional outcome both directly (estimate = −0.45, *p* < 0.001) and indirectly by reducing the occurrence of symptomatic intracranial hemorrhage (sICH) (estimate = −0.09, *p* = 0.004). This model explained 36.5% of the variance in 90-day mRS scores.

**Conclusion:**

In patients with acute ICAS-related MCA occlusion, stenting with angioplasty and achieving mTICI 2c–3 recanalization are associated with improved clinical outcome. These benefits are mediated by better post-procedural ASPECTS and reduced sICH. Prospective studies are warranted to confirm these findings.

**Supplementary Information:**

The online version of this article (10.1007/s00062-025-01577-6) contains supplementary material, which is available to authorized users.

## Introduction

Mechanical thrombectomy (MT) has become the standard of care for acute large vessel occlusion (LVO), demonstrating significantly higher recanalization rates compared to medical therapy alone [1]. However, MT is unsuccessful in 10–20% of patients, often due to challenging vascular anatomy that prevents access to the target vessel, the presence of a calcified clot, or underlying intracranial artery stenosis (ICAS) [2–5]. In cases of ICAS-LVO, rescue therapies, such as stent placement or angioplasty, serve as potential adjunctive maneuvers to address the underlying stenosis, thereby enhancing the likelihood of sustained recanalization and reducing the risk of re-occlusion [6]. Retrospective and registry studies have demonstrated that rescue stenting (RS) is associated with better angiographic results and improved clinical outcomes [7–9], particularly in cases where MT alone fails to achieve successful recanalization [10, 11]. On the other hand, a recent trial failed to demonstrate a clear benefit of balloon angioplasty with or without RS in patients with unsuccessful recanalization or residual stenosis > 70% following MT, compared to those treated with MT alone [12]. Thus, RS for acute ICAS-LVO remains a debated issue due to associated risks and the lack of data from randomized trials [13].

Here we report a retrospective multicenter analysis on patients with ICAS-LVO of the M1 segment of the middle cerebral artery (MCA) who received MT with RS. The purpose of this study was to define the determinants of functional outcome in this patient population, by evaluating the role of both baseline characteristics and procedural and post-procedural factors.

## Methods

### Patients and Treatment

This retrospective observational study involved 27 comprehensive stroke centers across Europe, United States and China. The study protocol was approved by the ethics committee of the coordinator center (protocol number 6410/20, ID 3004). Local ethics committees at each participating site also approved the use of patients’ data. Informed consent was waived due to the retrospective nature of the study and because all therapeutic procedures were part of the standard care.

The analysis was conducted in adherence with the STrengthening the Reporting of OBservational studies in Epidemiology (STROBE) statement.

Prospective databases at each center were screened for consecutive patients with acute MCA occlusion who received endovascular treatment between January 2020 and June 2024. All patients were initially assessed with a non-contrast computed tomography (CT), followed by CT angiography to locate the site of occlusion. Demographic data, cardiovascular risk factors, prior therapies, and baseline clinical/imaging data were collected. Intravenous thrombolysis (IVT) was administered in accordance with standard eligibility protocols. Mechanical thrombectomy was performed with a stent-retriever, direct contact aspiration or using a combined technique. Patients with underlying ICAS in the M1 segment of the MCA, identified during MT and subsequently treated with RS, were included in the study. Intracranial artery stenosis was defined as a 50–99% residual fixed stenosis or immediate re-occlusion not attributable to dissection or vasospasm after MT, according to previously published criteria [7, 10]. The decision to proceed with RS was left to the discretion of the treating neurointerventionalist. The RS technique included angioplasty performed either before or after stenting, depending on the operator’s preference, the angiographic appearance of the occlusion, and the devices available at the center. Successful recanalization was the modified Treatment In Cerebral Infarction (mTICI) score ≥ 2b at the end of the endovascular procedure. Time metrics, anesthesia modality, whether stenting was performed with or without angioplasty, and peri-procedural antiplatelet therapies were also recorded. Post-procedural outcomes, including the Alberta Stroke Program Early CT score (ASPECTS) and the occurrence of intracranial hemorrhage, were evaluated using follow-up CT imaging performed 24–72 h after the intervention. A symptomatic intracranial hemorrhage (sICH) was defined as any parenchymal hematoma associated with an increase of ≥ 4 points in the National Institutes of Health Stroke Scale (NIHSS) or ≥ 2 points in an NIHSS subcategory, according to the Heidelberg bleeding classification [14]. Radiological and angiographic data were reviewed locally by the neuroradiologists/neurointerventionalists blinded to clinical information.

### Outcome Measure

The outcome measure of interest was the 90-day functional status, assessed using the modified Rankin Scale (mRS) and analyzed as an ordinal variable.

## Statistical Analysis

Descriptive statistics were used to summarize baseline characteristics. Continuous variables were reported as medians with interquartile ranges (IQRs). Categorical variables were reported as counts and percentages. Fisher’s exact test was used to compare categorical variables. Continuous variables were compared using either the Welch two-sample t‑test or Mann-Whitney U test, depending on their distribution as determined by the Shapiro-Wilk test. Missing data were not imputed. Significance threshold was set at *p* < 0.05 for all analyses.

Stepwise multivariate regression analysis was conducted to identify predictors of outcome variables. Variables with *p* ≤ 0.1 in univariate analyses were included in the multivariate models. For continuous outcome variables, linear regression was used to calculate unstandardized estimates with standard errors (SEs) and 95% confidence intervals (CIs). For binary outcome variables, odds ratios (ORs) and their corresponding 95% CIs were calculated using logistic regression.

Structural equation modeling (SEM) was employed to overcome limitations of standard regression analysis by testing a conceptual model of causal pathways. Variables retained in the multivariate models and showing a significant *p*-value, were tested as predictors or mediators in SEM. The model was estimated using the weighted least squares mean and variance adjusted estimator for ordinal variables, providing regression coefficients that quantifies the direction and magnitude of effect on the ordinal mRS outcome. Model fit was assessed using the chi-square test, the comparative fit index (CFI), the root mean square error of approximation (RMSEA) and the standardized root mean square residual (SRMQR). Acceptable model fit was indicated by a non-significant chi-square test, CFI > 0.95, RMSEA < 0.06, and SRMQR < 0.08. Unstandardized estimates, 95% CIs and explained variances were extracted for each model. Indirect effects were evaluated using non-parametric bootstrapping to generate bias-corrected 95% CIs, allowing for robust estimation of mediation effects. All analyses were performed using R software v. 4.3.2, employing the *lavaan* package (https://www.r-project.org).

## Results

A total of 18,732 patients were screened, and 530 (28%) patients who underwent endovascular treatment for MCA ICAS-LVO were identified. Of these, 244 (13%) were treated with RS, with or without angioplasty. After excluding patients with occlusions outside the M1 segment of the MCA or those with missing baseline, procedural and post-procedural data, 115 patients (45 females, 39.1%) were included in the final analysis. A flow diagram of patient selection is presented in Supplementary Fig. 1.

Sixty-five patients (56.5%) received stenting with angioplasty. Successful recanalization at the end of the endovascular procedure was achieved in 94.8% of patients, with 73.0% reaching an mTICI 2c–3 score. Symptomatic intracranial hemorrhage occurred in 13% of patients. The median (IQR) 90-day mRS score was 3 (1–4) and patients achieving a 90-day mRS score 0–2 were 44.3%. Demographics and relevant clinical, radiological and procedural data, including metrics and periprocedural antiplatelet therapies, are summarized in Supplementary Table 1.

Univariate analysis for predictors of the 90-day mRS score is reported in Supplemental Material Table 2. In the stepwise linear regression model, baseline factors associated with worse 90-day mRS score included higher age, presence of coronary artery disease, and lower baseline ASPECTS. Post-procedural predictors of poor outcome were lower post-procedural ASPECTS and the occurrence of sICH (Table 1, 2 and 3).

**Table 1:** Stepwise multivariate regression analysis—predictors of 90-day mRS score

Variable	Estimate	SE	95% CI	p*
*Baseline data*				
Age	0.04	0.01	0.01– 0.07	*0.003*
Coronary artery disease	1.03	0.44	0.16– 1.91	*0.021*
Baseline ASPECTS	−0.30	0.12	−0.53– −0.06	*0.013*
*Post-procedural data*				
Post-procedural ASPECTS	−0.45	0.08	−0.61– −0.29	*<* *0.001*
sICH	1.60	0.44	0.72–2.50	*<* *0.001*

**Table 2:** Stepwise multivariate regression analysis—predictors of post-procedural ASPECTS

Variable	Estimate	SE	95% CI	p*
Baseline ASPECTS	0.63	0.11	0.42– 0.84	*<* *0.001*
GTR time	−0.004	0.002	−0.01– 0.005	0.053
Stent with angioplasty	0.78	0.29	0.19– 1.36	*0.010*
Final mTICI score 2c–3	0.70	0.33	0.04– 1.35	*0.037*

**Table 3:** Stepwise multivariate regression analysis—predictors of sICH

Variable	OR	95% CI	p*
Sex (female)	0.31	0.03– 1.01	0.165
Dyslipidemia	0.19	0.23– 1.00	0.065
Post-procedural ASPECTS	0.31	0.06– 0.85	*0.005*

*mRS* modified Rankin Scale; *SE* standard error; *CI* confidence interval; *ASPECTS* Alberta Stroke Program Early CT score; *sICH* symptomatic intracranial hemorrhage; *GTR* groin-to-reperfusion; *mTICI* modified Treatment In Cerebral Infarction

*significance set at *p* < 0.05

Using the same approach, we analyzed predictors of post-procedural ASPECTS and sICH. Univariate analyses are provided in Supplementary Tables 3 and 4. Regression analysis showed that, in addition to baseline ASPECTS, rescue therapy consisting in stenting with angioplasty and a final mTICI score 2c–3 were positively associated with post-procedural ASPECTS. A lower post-procedural ASPECTS was the only factor associated with increased risk of sICH (Table 1, 2 and 3).

Given the relationship between post-procedural ASPECTS, sICH and clinical outcome, we used SEM to test two hypotheses: 1) whether a rescue treatment using stenting with angioplasty and achieving a final mTICI 2c–3 score have an effect on clinical outcome that is mediated by post-procedural ASPECTS; 2) the association of post-procedural ASPECTS with better outcome is partially explained by a reduction of sICH risk.

Structural equation modeling showed that post-procedural ASPECTS served as a mediator of clinical outcome, influencing the 90-day mRS score both directly (estimate = −0.45, 95% CI −0.61 to −0.29, *p* < 0.001) and indirectly through reduction of sICH occurrence (estimate = −0.09, 95% CI −0.16 to −0.03, *p* = 0.004) (Table 4).

**Table 4:** Mediation analysis for 90-day mRS score

	Estimate	SE	95% CI	p*
*Effects of post-procedural ASPECTS on 90-day mRS score*				
Direct: post-procedural ASPECTS → 90-day mRS score	−0.45	0.08	−0.61– −0.29	*<* *0.001*
Indirect: post-procedural ASPECTS → sICH → 90-day mRS score	−0.09	0.03	−0.16– −0.03	*0.004*
*Baseline and procedural features* *→* *post-procedural ASPECTS* *→* *90-day mRS score*				
Baseline ASPECTS	−0.31	0.07	−0.45– −0.17	*<* *0.001*
Stent with angioplasty	−0.36	0.16	−0.67– −0.05	*0.02*
Final mTICI 2c–3 score	−0.35	0.18	−0.70– −0.01	*0.04*
*Baseline and procedural features* *→* *post-procedural ASPECTS* *→* *sICH* *→* *90-day mRS score*				
Baseline ASPECTS	−0.06	0.02	−0.10– −0.02	*0.01*
Stent with angioplasty	−0.07	0.03	−0.13– −0.01	*0.03*
Final mTICI 2c–3 score	−0.07	0.04	−0.15– 0.01	0.1

*mRS* modified Rankin Scale; *SE* standard error; *CI* confidence interval; *ASPECTS* Alberta Stroke Program Early CT score; *sICH* symptomatic intracranial hemorrhage; *mTICI* modified Treatment In Cerebral Infarction

*significance set at *p* < 0.05

**Fig. 1:**
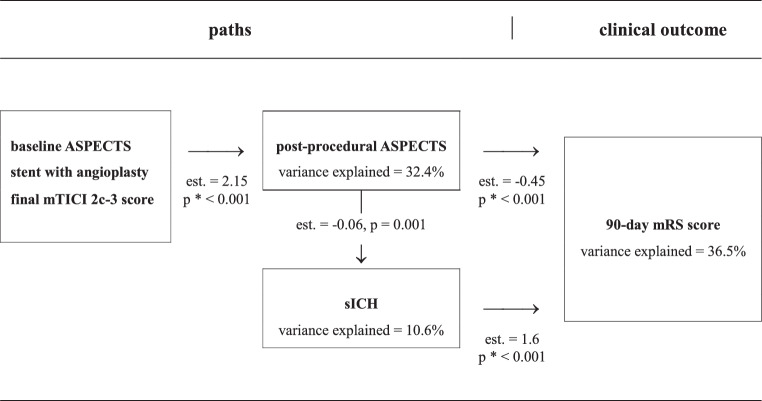
Comprehensive structural equation model and results overview. *ASPECTS* Alberta Stroke Program Early CT score; *mTICI* modified Treatment in Cerebral Infarction; est. estimate; *sICH* symptomatic intracranial hemorrhage; *mRS* modified Rankin Scale; ^*^significance set at *p* < 0.05

Baseline ASPECTS, stent with angioplasty and the final mTICI 2c–3 score showed a cumulative positive effect on post-procedural ASPECTS (estimate = 2.15, 95% CI 1.16, 3.14, *p* < 0.001) and accounted for 32.4% of its variance (Fig. 1). Stent with angioplasty was associated with a lower 90-day mRS score via post-procedural ASPECTS, both directly and, to a lesser extent, indirectly through sICH reduction. Achieving a final mTICI 2c–3 score improved clinical outcome via ASPECTS preservation, with no significant influence from sICH reduction. Detailed results of SEM analysis are reported in Table 4. Notably, the effect of mTICI 2c–3 recanalization on post-procedural ASPECTS did not differ by stent-with-angioplasty status (interaction estimate = −1.22, 95% CI −2.75 to 0.31, *p* = 0.12), suggesting separate mechanisms. The full model (effect of baseline and treatment variables on clinical outcome through post-procedural ASPECTS and sICH occurrence) explained 36.9% of the variance in 90-day mRS score (Fig. 1). This increased to 40% after adjusting for age and presence of coronary artery disease (Supplementary Fig. 2). The protective effect of post-procedural ASPECTS on sICH occurrence accounted for 10.6% of its variance (Fig. 1). Model fit indices are reported in Supplementary Table 5.

## Discussion

Our study has shown that stenting with angioplasty and achieving high-quality recanalization improve post-procedural ASPECTS, which directly mediates functional benefit and lowers sICH risk. This aligns with prior evidence that post-procedural ASPECTS is a critical predictor of outcome and hemorrhage, and that the benefit of stenting is mediated by limiting infarct progression [7, 15]. Post-procedural ASPECTS explained a relatively small proportion (10.6%) of sICH variance, suggesting that the primary mechanisms of hemorrhagic complications in ICAS-LVO remain largely unknown. No clear association was found between sICH and peri-procedural antithrombotic therapies or IVT. This finding aligns with studies showing rescue stenting does not significantly increase sICH risk versus MT alone, suggesting it is not an exclusively treatment-related complication [7, 10, 12]. Nonetheless, the optimal peri-procedural antiplatelet strategy remains uncertain and warrants prospective investigation.

While both stenting alone and stenting with angioplasty have shown a favorable effect on clinical outcome compared to MT only, data directly comparing these two technical strategies remain limited [7, 16]. Recently, a post-hoc analysis from the Stenting and Angioplasty in NeuroThrombectomy (SAINT) study has showed that patients treated with balloon mounted stent had higher reperfusion rates than with self-expanding stent [16]. Retrospective studies and meta-analyses in patients with symptomatic ICAS have shown that the combination of angioplasty and stenting is associated with lower rates of in-stent restenosis, stroke recurrence, and mortality compared to stenting alone [17–19]. In the acute setting, the study by Stracke and colleagues demonstrated that balloon angioplasty followed by deployment of a self-expanding stent yielded outcomes that were within or even better than the ranges reported in earlier studies using self-expandable stents without angioplasty [20–22]. By contrast, the Randomised trial of Bailout intracranial angioplasty or stenting following Thrombectomy for acute large vessel occlusion (ANGEL-REBOOT) trial found no overall functional benefit of bailout angioplasty or stenting after thrombectomy, although the effects of specific rescue strategies were not analyzed and outcomes in the control arm were unexpectedly favorable [12]. Our analysis supports that the combination of angioplasty and stenting is instead beneficial. It is conceivable that the combined treatment may limit the elastic recoil of the vessel or an incomplete stent expansion, thus sustaining the effect of immediate recanalization with improved downstream flow and capillary perfusion. However, stent with angioplasty is not without risk, as dissection, vessel perforation or plaque disruption with distal downstream embolization remain as potential concerns. Its feasibility should be carefully considered, with angioplasty potentially enhancing stent expansion and durability in high-grade or MT-refractory stenoses without complex vascular anatomy. Careful patient selection, procedural planning, and operator expertise are therefore essential. In this context, relevant information will likely be provided by the ongoing Permanent Intracranial STenting for Acute Refractory large vessel occlusions (PISTAR) and the angioplasty and/or stenting following successful mechanical thrombectomy for intracranial atherosclerosis-related emergent large vessel occlusive stroke (ASSET) randomized clinical trials [23, 24].

Our results also highlight the clinical value of achieving mTICI 2c–3 recanalization grade at the end of the rescue endovascular procedure rather than settling for 2b only [25–27]. Indeed, although mTICI 2b is often considered a satisfactory endpoint, our analysis found that it was not associated with meaningful improvements of post-procedural ASPECTS or clinical outcome. Currently, there is a lack of studies comparing the outcomes of mTICI 2b versus mTICI 2c–3 recanalization grades in patients with acute ICAS-LVO after RS. Although in certain clinical scenarios the pursuit of higher recanalization grades might not confer additional benefits [28], our findings show that in ICAS-LVO patients, achieving near-complete or complete recanalization more effectively limits infarct progression and improves clinical outcome. This may be due to the incomplete reperfusion associated with a 2b score, which can leave critical regions, such as those supplied by the lenticulostriate arteries, insufficiently perfused. Subtle differences in recanalization quality may therefore translate into marked differences in infarct progression or collateral flow dynamics. This may help also explain why previous studies failed to show improvements in 90-day mRS scores in patients receiving adjunct rescue therapy compared to MT alone, despite higher recanalization rates primarily driven by mTICI 2b outcomes [29].

## Limitations

The main limitation of our study is its retrospective, non-controlled design, which introduces potential selection bias. Data quality constraints inherent to non-randomized studies and local imaging assessments may have influenced the results. Variability in endovascular protocols across centers, operator-dependent decisions regarding rescue stenting and adjunctive angioplasty, and the heterogeneity of devices (including both coronary and neurovascular stents) represent additional sources of bias. The recorded use of diverse peri-procedural antiplatelet therapies (including single agents, dual antiplatelets, and glycoprotein IIb/IIIa inhibitors alone or in combination) limits the ability to explore their potential effects on outcomes in this relatively small cohort. Moreover, applying SEM in a relatively small sample carries the risk of overfitting, highlighting the need for validation in larger prospective studies before integrating these findings into predictive models. Finally, our analysis was restricted to patients treated with rescue stenting, either with or without adjunctive angioplasty, which precludes direct comparison with alternative rescue strategies.

## Conclusions

In patients with acute M1-MCA LVO due to underlying ICAS, stenting with angioplasty and achieving an mTICI 2c–3 recanalization grade are associated with better clinical outcome. These benefits appear to be mediated by improved post-procedural ASPECTS, which directly enhances functional recovery and reduces the risk of sICH. Prospective studies are warranted to confirm and further explore these findings.

## Supplementary Information

Supplementary Information includes the flow diagram of patient selection, all baseline, procedural, and outcome data of the patient population, the results of all univariate analyses, and the comprehensive Structural Equation Modeling that incorporates the effects of age and coronary artery disease
