# Supplementary material for: Impact of Stenting with Angioplasty and MTICI 2c-3 Recanalization On Outcome in Acute MCA Occlusion with Underlying Stenosis
Source: Clin Neuroradiol. 2025 Oct 13;36(2):329–37. doi: 10.1007/s00062-025-01577-6 (PMC13320014; doi:10.1007/s00062-025-01577-6)
Supplement: Supplementary file 1 — Supplementary Information includes the flow diagram of patient selection, all baseline, procedural, and outcome data of the patient population, the results of all univariate analyses, and the comprehensive Structural Equation Modeling that incorporates the effects of age and coronary artery disease [file 62_2025_1577_MOESM1_ESM.docx]

**Impact of stenting with angioplasty and mTICI 2c-3 recanalization on outcome in acute MCA occlusion with underlying stenosis**

**Supplementary Material**

**Supplementary Figure 1**

**
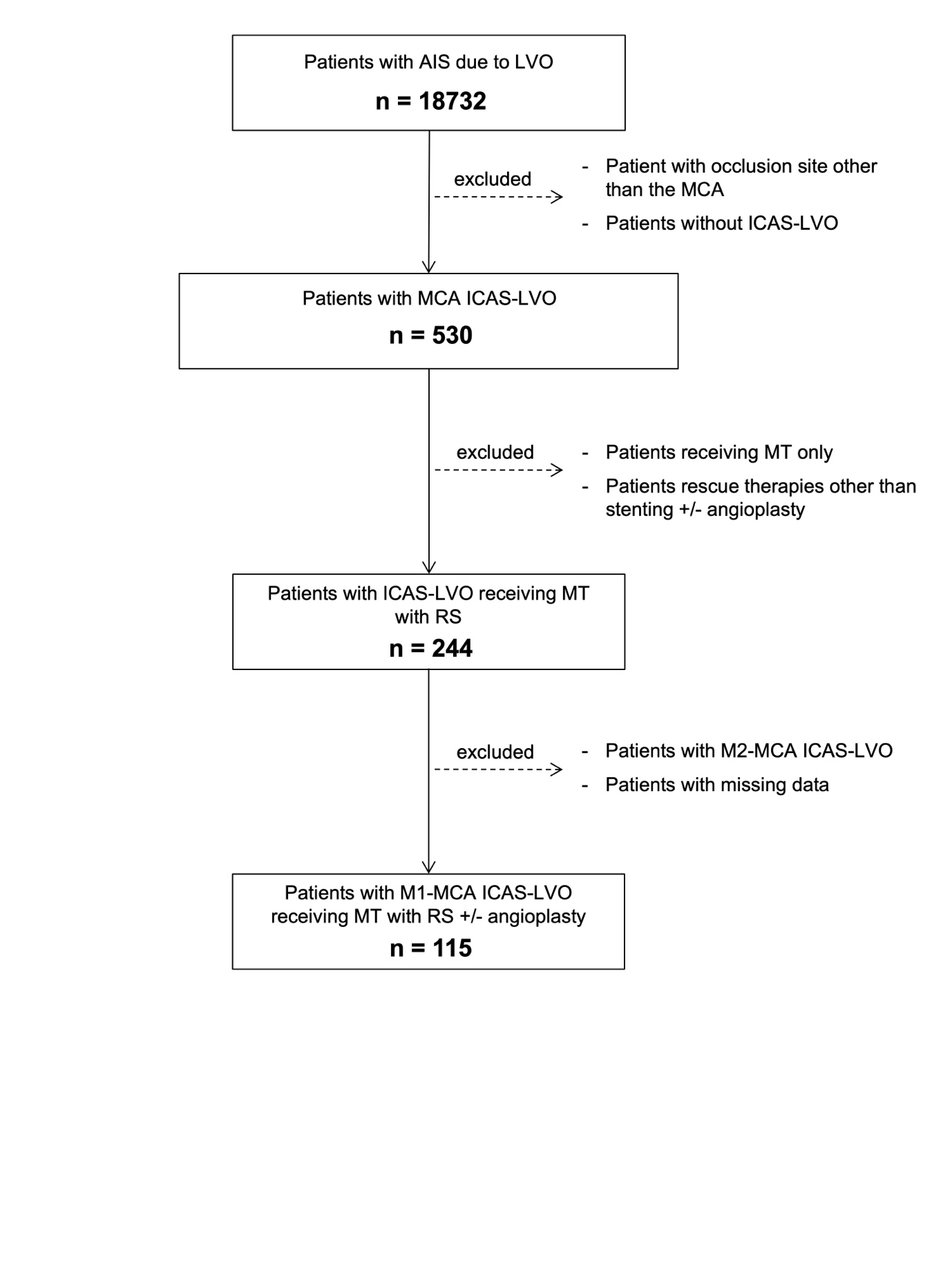
**

**Supplementary Table 1. Demographics, baseline clinical, procedural and outcome data of patients**

|  |  | | |  |
| --- | --- | --- | --- | --- |
| **variable** |  | | | **patients (N = 115)** |
|  |  | | |  |
|  |  | | |  |
| **demographics, baseline clinical and radiological features** | | | |  |
| age (years), median (IQR) |  | | | 69 (59-76) |
| sex (female), n/N (%) |  | | | 45/115 (39.1) |
| hypertension, n/N (%) |  | | | 83/115 (72.2) |
| dyslipidemia, n/N (%) |  | | | 44/115 (38.3) |
| diabetes, n/N (%) |  | | | 31/115 (27.0) |
| atrial fibrillation, n/N (%) |  | | | 18/115 (15.7) |
| coronary artery disease, n/N (%) |  | | | 19/115 (16.5) |
| current smoker, n/N (%) |  | | | 12/97 (12.4) |
| prior antiplatelet therapy, n/N (%) |  | | | 27/115 (23.5) |
| prior anticoagulant therapy, n/N (%) |  | | | 14/115 (12.2) |
| prior therapy with statins, n/N (%) |  | | | 31/115 (27.0) |
| pre-event mRS score, median (IQR) |  | | | 0 (0-0) |
| baseline NIHSS score, median (IQR) |  | | | 14 (10-18) |
| left-side stroke, n/N (%) |  | | | 58/115 (50.4) |
| baseline ASPECTS, median (IQR) |  | | | 9 (8-10) |
|  |  | | |  |
| **procedural data** |  | | |  |
| IVT, n/N (%) |  | | | 28/115 (24.3) |
| general anesthesia, n/N (%) |  | | | 63/115 (54.8) |
| OTG time (minutes), median (IQR) |  | | | 342 (210-600) |
| GTR time (minutes), median (IQR) |  | | | 80 (50-120) |
|  |  | | |  |
| degree of MCA stenosis, n/N (%) |  | | |  |
| 50-75% |  | | | 24/115 (20.9) |
| 75-99% |  | | | 69/115 (60.0) |
| 100% |  | | | 22/115 (19.1) |
|  |  | | |  |
| number of passes, median (IQR) |  | | | 2 (1-3) |
| stent with angioplasty, n/N (%) |  | | | 65/115 (56.5) |
|  |  | | |  |
| type of peri-procedural antiplatelets agent, n/N (%) | |  |  | |
| aspirin |  | | | 15/110 (13.6) |
| cangrelor |  | | | 8/110 (7.3) |
| GPI |  | | | 43/110 (39.1) |
| clopidogrel |  | | | 4/110 (3.6) |
| aspirin + clopidogrel |  | | | 7/110 (6.4) |
| aspirin + GPI |  | | | 13/110 (11.8) |
| aspirin + clopidogrel + GPI |  | | | 11/110 (10.0) |
| clopidogrel + GPI |  | | | 3/110 (2.7) |
| aspirin + cangrelor |  | | | 6/110 (5.5) |
|  |  | | |  |
| final mTICI score, n/N (%) |  | | |  |
| 0-1 |  | | | 3/115 (2.6) |
| 2a |  | | | 3/115 (2.6) |
| 2b |  | | | 25/115 (21.7) |
| 2c-3 |  | | | 84/115 (73.1) |
|  |  | | |  |
| **post-procedural data** |  | | |  |
| post-procedural ASPECTS |  | | | 7 (6-8) |
| sICH |  | | | 15/115 (13.0) |
|  |  | | |  |
| **clinical outcome** |  | | |  |
| 90-day mRS score, median (IQR) |  | | | 3 (1-4) |
| patients achieving 90-day mRS score 0-2, n/N (%) |  | | | 51/115 (44.3) |
|  |  | | |  |

IQR, interquartile range; mRS, modified Rankin scale; NIHSS, National Institutes of Health Stroke Scale; ASPECTS, Alberta Stroke Program Early CT score; IVT, intravenous thrombolysis; OTG, onset-to-groin; GTR, groin-to-reperfusion; MCA, middle cerebral artery; GPI, Glycoprotein IIb/IIIa inhibitor; mTICI, modified Treatment in Cerebral Infarction; sICH, symptomatic intracranial hemorrhage.

**Supplementary Table 2. Univariate analysis for predictors of 90-day mRS score**

|  |  | |  | |  |  |
| --- | --- | --- | --- | --- | --- | --- |
| **variable** | **estimate** | | **95% CI** | | **p*** |  |
|  |  | |  | |  |  |
|  |  | |  | |  |  |
| **demographics, baseline clinical and radiological features** | | |  | |  |  |
| age | 0.04 | | 0.01 – 0.1 | | **0.008** |  |
| sex (female) | -0.06 | | -0.8 – 0.7 | | 0.9 |  |
| hypertension | 0.6 | | -0.2 – 1.4 | | 0.1 |  |
| dyslipidemia | -0.2 | | -0.9 – 0.5 | | 0.6 |  |
| diabetes | 0.4 | | -0.3 – 1.2 | | 0.3 |  |
| atrial fibrillation | 0.3 | | -1.2 – 0.7 | | 0.6 |  |
| coronary artery disease | 1.2 | | 0.3 – 2.1 | | **0.01** |  |
| current smoker | -0.4 | | -1.5 – 0.8 | | 0.5 |  |
| prior antiplatelet therapy | 0.6 | | -0.3 – 1.4 | | 0.2 |  |
| prior anticoagulant therapy | 0.8 | | -0.3 – 1.8 | | 0.2 |  |
| prior therapy with statins | 0.04 | | -0.7 – 0.8 | | 0.9 |  |
| pre-event mRS score | 0.5 | | -0.1 – 1.0 | | 0.1 |  |
| baseline NIHSS score | 0.06 | | -0.003 – 0.1 | | 0.06 |  |
| left-side stroke | -0.3 | | -1.0 – 0.4 | | 0.4 |  |
| baseline ASPECTS | -0.3 | | -0.5 – -0.03 | | **0.03** |  |
|  |  | |  | |  |  |
| **procedural data** |  | |  | |  |  |
| IVT | -0.1 | | -0.9 – 0.7 | | 0.8 |  |
| general anesthesia | 0.5 | | -0.2 – 1.2 | | 0.1 |  |
| OTG time | 0.0002 | | -0.0005 – 0.001 | | 0.5 |  |
| GTR time | 0.0008 | | -0.004 – 0.006 | | 0.8 |  |
|  |  | |  | |  |  |
| degree of MCA stenosis |  | |  | | 0.1 |  |
| 50-75% (reference) |  | |  | |  |  |
| 75-99% | -0.9 | | -1.8 – -0.02 | | **0.04** |  |
| 100% | -0.3 | | -1.6 – 0.6 | | 0.4 |  |
|  |  | |  | |  |  |
| number of passes | 0.1 | | -0.2 – 0.3 | | 0.6 |  |
| stent with angioplasty | -0.5 | | -1.2 – 0.2 | | 0.2 |  |
| use of peri-procedural antiplatelets agents | 0.08 | | -1.5 – 1.6 | | 0.9 |  |
|  |  | |  | |  |  |
| type of peri-procedural antiplatelets agent | |  | |  | 0.7 | |
| aspirin (reference) |  | |  | |  | |
| cangrelor | 1.6 | | -0.01 – 3.3 | | 0.05 | |
| GPI | 0.4 | | -0.8 – 1.5 | | 0.5 | |
| clopidogrel | 0.2 | | -2.0 – 2.3 | | 0.9 | |
| aspirin + clopidogrel | 0.7 | | -1.0 – 2.4 | | 0.4 | |
| aspirin + GPI | 0.1 | | -1.3 – 1.5 | | 0.9 | |
| aspirin + clopidogrel + GPI | -0.2 | | -1.7 – 1.2 | | 0.8 | |
| clopidogrel + GPI | 0.4 | | -2.0 – 2.8 | | 0.7 | |
| aspirin + cangrelor | -0.3 | | -2.1 – 1.6 | | 0.7 | |
|  |  | |  | |  |  |
| final mTICI score |  | |  | | 0.8 |  |
| 0-1 (reference) |  | |  | |  |  |
| 2a | -0.7 | | -3.7 – 2.4 | | 0.7 |  |
| 2b | -0.5 | | -2.8 – 1.8 | | 0.7 |  |
| 2c-3 | -0.9 | | -3.1 – 1.3 | | 0.4 |  |
|  |  | |  | |  |  |
| **post-procedural data** |  | |  | |  |  |
| post-procedural ASPECTS | -0.5 | | -0.7 – -0.4 | | **<0.001** |  |
| sICH | 2.4 | | 1.5 – 3.4 | | **<0.001** |  |
|  |  | |  | |  |  |

mRS, modified Rankin scale; CI, confidence interval; NIHSS, National Institutes of Health Stroke Scale; ASPECTS, Alberta Stroke Program Early CT score; IVT, intravenous thrombolysis; OTG, onset-to-groin; GTR, groin-to-reperfusion; MCA, middle cerebral artery; GPI, Glycoprotein IIb/IIIa inhibitor; mTICI, modified Treatment in Cerebral Infarction; sICH, symptomatic intracranial hemorrhage; * significance set at p < 0.05.

**Supplementary Table 3. Univariate analysis for predictors of post-procedural ASPECTS**

|  |  |  |  |  |
| --- | --- | --- | --- | --- |
| **variable** | **estimate** | **95% CI** | **p*** |  |
|  |  |  |  |  |
|  |  |  |  |  |
| **demographics, baseline clinical and radiological features** | |  |  |  |
| age | -0.003 | -0.03 – 0.03 | 0.9 |  |
| sex | 0.6 | -0.1 – 1.3 | 0.08 |  |
| hypertension | -0.3 | -1.1 – 0.5 | 0.5 |  |
| dyslipidemia | 0.3 | -0.4 – 1.0 | 0.4 |  |
| diabetes | -0.2 | -1.1 – 0.5 | 0.5 |  |
| atrial fibrillation | 0.4 | -0.6 – 1.3 | 0.4 |  |
| coronary artery disease | -0.6 | -1.6 – 0.3 | 0.2 |  |
| current smoker | -0.6 | -1.8 – 0.5 | 0.3 |  |
| prior antiplatelet therapy | -0.01 | -0.8 – 0.8 | 0.9 |  |
| prior anticoagulant therapy | -0.3 | -1.3 – 0.8 | 0.6 |  |
| prior therapy with statins | 0.3 | -0.4 – 1.1 | 0.4 |  |
| pre-event mRS score | 0.2 | -0.4 – 0.7 | 0.6 |  |
| baseline NIHSS score | -0.05 | -0.1 – 0.01 | 0.09 |  |
| left-side stroke | 0.1 | -0.6 – 0.8 | 0.7 |  |
| baseline ASPECTS | 0.8 | 0.6 – 0.9 | **<0.001** |  |
|  |  |  |  |  |
| **procedural data** |  |  |  |  |
| IVT | 0.1 | -0.7 – 0.9 | 0.7 |  |
| general anesthesia | -0.7 | -1.4 – 0.01 | **0.046** |  |
| OTG time | -0.0003 | -0.001 – 0.005 | 0.5 |  |
| GTR time | -0.0005 | -0.001 – 0.002 | **0.04** |  |
|  |  |  |  |  |
| degree of MCA stenosis |  |  | 0.8 |  |
| 50-75% (reference) |  |  |  |  |
| 75-99% | 0.3 | -0.6 – 1.2 | 0.5 |  |
| 100% | 0.1 | -0.9 – 1.2 | 0.7 |  |
|  |  |  |  |  |
| number of passes | -0.2 | -0.4 – 0.01 | 0.06 |  |
| stent with angioplasty | 0.7 | 0.01 – 1.4 | **0.04** |  |
| use of peri-procedural antiplatelets agents | 1.4 | -0.1 – 2.9 | 0.07 |  |
|  |  |  |  |  |
| type of peri-procedural antiplatelets agent |  |  | 0.3 | |
| aspirin (reference) |  |  |  | |
| cangrelor | -0.9 | -2.7 – 0.7 | 0.3 | |
| GPI | 0.03 | -1.1 – 1.1 | 0.9 | |
| clopidogrel | 0.7 | -1.3 – 2.8 | 0.5 | |
| aspirin + clopidogrel | -0.4 | -2.1 – 1.3 | 0.6 | |
| aspirin + GPI | -0.3 | -1.7 – 1.1 | 0.6 | |
| aspirin + clopidogrel + GPI | 0.4 | -1.1 – 1.8 | 0.6 | |
| clopidogrel + GPI | -0.6 | -2.9 – 1.7 | 0.6 | |
| aspirin + cangrelor | 1.1 | -0.7 – 2.8 | 0.2 | |
|  |  |  |  |  |
| final mTICI score |  |  | **0.049** |  |
| 0-1 (reference) |  |  |  |  |
| 2a | 2.3 | -0.6 – 5.3 | 0.1 |  |
| 2b | 1.6 | -0.6 – 3.8 | 0.2 |  |
| 2c-3 | 2.4 | 0.3 – 4.6 | **0.03** |  |
|  |  |  |  |  |

ASPECTS, Alberta Stroke Program Early CT score; CI, confidence interval; mRS, modified Rankin scale; NIHSS, National Institutes of Health Stroke Scale; IVT, intravenous thrombolysis; OTG, onset-to-groin; GTR, groin-to-reperfusion; MCA, middle cerebral artery; GPI, Glycoprotein IIb/IIIa inhibitor; mTICI, modified Treatment in Cerebral Infarction; * significance set at p < 0.05.

**Supplementary Table 4. Univariate analysis for predictors of sICH**

|  |  |  |  |
| --- | --- | --- | --- |
| **variable** | **sICH**  **(N = 15)** | **no-sICH**  **(N = 100)** | **p*** |
|  |  |  |  |
|  |  |  |  |
| **demographics, baseline clinical and radiological features** | |  |  |
| age, median (IQR) | 73 (63-75) | 67 (59-76) | 0.4 |
| sex (female), n/N (%) | 2/15 (13.0) | 43/100 (43.0) | **0.03** |
| hypertension, n/N (%) | 12/15 (80.0) | 71/100 (71.0) | 0.5 |
| dyslipidemia, n/N (%) | 2/15 (13.0) | 42/100 (42.0) | **0.03** |
| diabetes, n/N (%) | 4/15 (26.7) | 37/100 (27.0) | 1.0 |
| atrial fibrillation, n/N (%) | 3/12 (20.0) | 15/100 (15.0) | 0.6 |
| coronary artery disease, n/N (%) | 4/15 (26.7) | 15/100 (15.0) | 0.2 |
| current smoker, n/N (%) | 0/11 (0.0) | 12/86 (12.9) | 0.2 |
| prior antiplatelet therapy, n/N (%) | 2/15 (13.0) | 25/100 (25.0) | 0.3 |
| prior anticoagulant therapy, n/N (%) | 3/12 (20.0) | 11/100 (11.0) | 0.3 |
| prior therapy with statins, n/N (%) | 3/12 (20.0) | 28/100 (28.0) | 0.5 |
| pre-event mRS score, median (IQR) | 0 (0-1) | 0 (0-0) | 0.4 |
| baseline NIHSS score, median (IQR) | 15 (10-19) | 14 (10-18) | 0.9 |
| left-side stroke, n/N (%) | 3/15 (20.0) | 55/100 (55.0) | **0.01** |
| baseline ASPECTS, median (IQR) | 9 (8-10) | 9 (8-10) | 0.9 |
|  |  |  |  |
| **procedural and post-procedural data** |  |  |  |
| IVT, n/N (%) | 6/15 (40.0) | 22/100 (22.0) | 0.13 |
| general anesthesia, n/N (%) | 10/15 (66.7) | 53/100 (53.0) | 0.3 |
| OTG time (minutes), median (IQR) | 200 (143-577) | 360 (240-600) | 0.2 |
| GTR time (minutes), median (IQR) | 70 (39-90) | 81 (50-120) | 0.3 |
|  |  |  |  |
| degree of MCA stenosis, n/N (%) |  |  | 0.4 |
| 50-75% | 5/15 (33.3) | 19/100 (19.0) |  |
| 75-99% | 7/15 (46.7) | 62/100 (62.0) |  |
| 100% | 3/15 (20.0) | 19/100 (19.0) |  |
|  |  |  |  |
| number of passes, median (IQR) | 2 (1-4) | 2 (1-3) | 0.6 |
| stent with angioplasty, n/N (%) | 9/15 (60.0) | 56/100 (56.0) | 0.8 |
| use of peri-procedural antiplatelets agents, n/N (%) | 15/15 (100) | 94/100 (94.0) | 0.3 |
|  |  |  |  |
| type of peri-procedural antiplatelets agent, n/N (%) |  |  | 0.5 |
| aspirin | 2/15 (13.3) | 13/100 (13.0) |  |
| cangrelor | 3/15 (20) | 5/100 (5.0) |  |
| GPI | 7/15 (46.6) | 36/100 (36.0) |  |
| clopidogrel | 0/15 (0) | 4/100 (4.0) |  |
| aspirin + clopidogrel | 0/15 (0) | 7/100 (7.0) |  |
| aspirin + GPI | 1/15 (6.6) | 12/100 (12.0) |  |
| aspirin + clopidogrel + GPI | 1/15 (6.6) | 10/100 (10.0) |  |
| clopidogrel + GPI | 0/15 (0) | 3/100 (3.0) |  |
| aspirin + cangrelor | 1/15 (6.6) | 5/100 (5.0) |  |
|  |  |  |  |
| final mTICI score, n/N (%) |  |  | 0.4 |
| 0-1 | 0/15 (0) | 3/100 (3.0) |  |
| 2a | 1/15 (6.7) | 2/100 (2.0) |  |
| 2b | 5/15 (33.3) | 20/100 (20.0) |  |
| 2c-3 | 9/15 (60.0) | 75/100 (75.0) |  |
|  |  |  |  |
| Post-procedural ASPECTS, median (IQR) | 6 (4-7) | 8 (6-9) | **<0.001** |
|  |  |  |  |

sICH, symptomatic intracranial hemorrhage; IQR, interquartile range; mRS, modified Rankin scale; NIHSS, National Institutes of Health Stroke Scale; ASPECTS, Alberta Stroke Program Early CT score; IVT, intravenous thrombolysis; OTG, onset-to-groin; GTR, groin-to-reperfusion; MCA, middle cerebral artery; GPI, Glycoprotein IIb/IIIa inhibitor; mTICI, modified Treatment in Cerebral Infarction; * significance set at p < 0.05.

**Supplementary Figure 2. Comprehensive structural equation model and results overview, after adjustment for age and coronary artery disease**

|  | | | | |
| --- | --- | --- | --- | --- |
| **paths** | | | ⏐ | **clinical outcome** |
|  | | | | |
|  |  |  |  |  |
|  |  |  |  |  |
|  |  |  |  |  |
| **age** | ⎯⎯⎯⎯⎯⎯⎯⎯⎯⎯⎯⎯⎯⎯⎯⎯⎯⎯→ | | |  |
|  | est. = 0.03, p * = 0.005 | | |  |
|  |  | | |  |
|  |  | | |  |
| **coronary artery disease** | ⎯⎯⎯⎯⎯⎯⎯⎯⎯⎯⎯⎯⎯⎯⎯⎯⎯⎯→ | | |  |
|  |  | est. = 0.73, p * = 0.06 |  |  |
|  |  |  |  | **90-day mRS score** |
|  |  |  |  | variance explained = 40.0% |
| **baseline ASPECTS** | ⎯⎯→ | **post-procedural ASPECTS** | ⎯⎯→ |  |
| **stent with angioplasty** | est. = 2.15 | variance explained = 32.4% | est. = -0.45 |  |
| **final mTICI 2c-3 score** | p * < 0.001 | ⏐ | p * < 0.001 |  |
|  |  | est. = -0.06, p * = 0.001 |  |  |
|  |  | ↓ |  |  |
|  |  |  |  |  |
|  |  | **sICH** | ⎯⎯→ |  |
|  |  | variance explained = 10.6% | est. = 1.6 |  |
|  |  |  | p * < 0.001 |  |
|  | | | | |
|  | | | | |

ASPECTS, Alberta Stroke Program Early CT score; mTICI, modified Treatment in Cerebral Infarction; est., estimate; sICH, symptomatic intracerebral hemorrhage; mRS, modified Rankin scale; *, significance set at p < 0.05.

**Supplementary Table 5. Fit indices of structural equation model**

|  |  |  |
| --- | --- | --- |
| **Fit index** | **value** | **threshold for good fit** |
|  |  |  |
|  |  |  |
| Chi-Square Test (χ²) | *p* = 0.63 | > 0.05 |
| CFI | 1.0 | > 0.95 |
| RMSEA | 0.0 | < 0.06 |
| SRMSR | 0.03 | < 0.08 |
|  |  |  |

mRS, modified Rankin Scale; CFI, Comparative Fit Index; RMSEA, Root Mean Square Error of Approximation; SRMSR, Standardized Root Mean Square Residual; ASPECTS, Alberta Stroke Program Early CT Score; sICH, symptomatic intracranial hemorrhage.
